# Novel Hantavirus in Wildlife, United Kingdom

**DOI:** 10.3201/eid1904.121057

**Published:** 2013-04

**Authors:** Kieran C. Pounder, Michael Begon, Tarja Sironen, Heikki Henttonen, Phillip C. Watts, Liina Voutilainen, Olli Vapalahti, Boris Klempa, Anthony R. Fooks, Lorraine M. McElhinney

**Affiliations:** University of Liverpool, Liverpool, UK (K.C. Pounder, M. Begon, P.C. Watts);; University of Helsinki, Helsinki, Finland (T. Sironen, L. Voutilainen, O. Vapalahti);; Finnish Forest Research Institute, Vantaa, Finland (H. Henttonen, L. Voutilainen);; Slovak Academy of Sciences, Bratislava, Slovakia (B. Klempa);; Charité School of Medicine, Berlin, Germany (B. Klempa);; Animal Health and Veterinary Laboratories Agency, Surrey, UK (A.R. Fooks, L.M. McElhinney);; University of Liverpool, National Consortium for Zoonosis Research, South Wirral, UK (A.R. Fooks, L.M. McElhinney)

**Keywords:** hantavirus, Tatenale, field vole, United Kingdom, viruses, wildlife

**To the Editor:** Hantaviruses (family *Bunyaviridae*) are transmitted to humans by inhalation of aerosolized virus in contaminated urine and feces, mainly from rodents of the families Cricetidae and Muridae. Although infections in rodents are asymptomatic, infections in humans can lead to hemorrhagic fever with renal syndrome and hantavirus cardiopulmonary syndrome (*1*).

In Europe, 5 rodent-borne hantaviruses have been detected: Dobrava-Belgrade, Saaremaa, Seoul, Puumala, and Tula (*1*,*2*). The most common and widespread hantavirus in Europe is Puumala virus, which is associated with the mildest form of hemorrhagic fever with renal syndrome (*1*).

In the United Kingdom, only a few cases of hantavirus infection in humans have been reported and confirmed serologically, but the causative virus species were not identified (*3*,*4*). Subsequent longitudinal studies reported considerable hantavirus seropositivity among healthy human cohorts, suggesting past exposure to hantaviruses or subclinical infection (*3*). Serologic surveys of rodents (rats and mice) and cats also supported the presence of a hantavirus indigenous to the United Kingdom (*3*). To determine whether hantaviruses are circulating in wild rodents in the United Kingdom, we conducted molecular analyses on rodent tissues. 

From September 2009 through November 2011, a total of 495 wild rodents consisting of 133 brown rats (*Rattus norvegicus*), 269 wood mice (*Apodemus sylvaticus*), 50 house mice (*Mus musculus*), 35 bank voles (*Myodes glareolus*), and 8 field voles (*Microtus agrestis*) were caught live across northwestern England (Technical Appendix). Animals were euthanized in the field by use of isoflurane inhalation, according to UK Home Office Guidelines (http://webarchive.nationalarchives.gov.uk/+/http://www.homeoffice.gov.uk/docs/hc193.html). Within 2 hours, kidney, liver, and lung tissues were removed. When field conditions allowed, blood samples were collected; otherwise, heart tissue was collected. Samples, and carcasses that could not be processed within 2 hours, were stored at –80°C. 

RNA was extracted by using TRIzol Reagent (Invitrogen, Life Technologies, Paisley, UK). To detect hantavirus RNA, we used a nested pan-hantavirus reverse transcription PCR selective for partial polymerase large segment (L) gene sequences (*5*). With the exception of 1 male field vole (B41) collected near Tattenhall, Cheshire (online Technical Appendix Figure), all lung samples were negative for hantavirus RNA. The positive amplicon was sequenced by using a BigDye Terminator 3.1v Cycle Sequencing Kit on an ABI3130xl genetic analyzer (Applied Biosystems/Life Technologies, Paisley, UK) (GenBank accession no. JX316008). Partial small segment (S) sequences were also recovered from lung RNA from vole B41 (GenBank accession no. JX316009) (Technical Appendix). Established reverse transcription PCRs for the medium segment were unsuccessful.

Comparisons of nucleotide and amino acid sequence identities demonstrated, as expected, that the Arvicolinae-associated hantaviruses showed the highest similarity to the UK sequence at the nucleotide (65.7%–78.8% for S and 76.6%–77.5% for L) and the amino acid (66.4%–86.3% for S and 80%–88% for L) levels (Technical Appendix).

Phylogenetic analyses of partial L (Figure, panel A) and partial S sequences (Figure, panel B) confirm the inclusion of the viral sequence from vole B41 as a distinct member of the Arvicolinae-associated hantaviruses. In the partial L tree (Figure, panel A), viral sequence B41 clustered with Prospect Hill and Tula viruses with good support, although in the partial S tree (Figure, panel B), B41 seems to be more closely related to the Asian *Microtus* vole–associated hantaviruses, albeit with low posterior probability values. These differences in tree topologies probably reflect different compositions of the sequence datasets.

**Figure:**
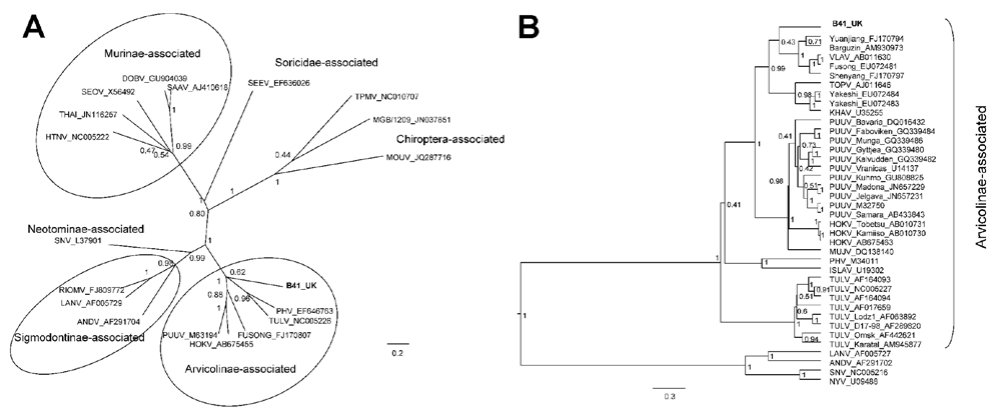
Bayesian phylogenetic trees constructed by using the models HKY+gamma for partial large segment sequences (n = 19) (A) and GTR+gamma for partial small segment sequences (n = 39) (B) within BEAST software (*6*) with Markov chain Monte Carlo chain lengths of 10 million and strict clock. Optimum substitution models were estimated by using MEGA5 (*7*). The trees are drawn to scale; branch lengths are measured in the number of substitutions per site. The numbers at each node are posterior probabilities. All effective sample size values exceeded 150 for partial L and 1,600 for partial S sequences. The phylogenetic position of virus isolated from field vole B41 (in boldface) is shown in relation to representative hantaviruses (A) and more closely related Arvicolinae-associated hantaviruses (B). GenBank accession numbers are shown next to taxonomic names. Scale bars indicate nucleotide substitutions per site. VLAV, Vladivostok virus; TOPV, Topografov virus; KHAV, Khabarovsk virus; PUUV, Puumala virus; HOKV, Hokkaido virus; MUJV, Muju virus; PHV, Prospect Hill virus; ISLAV, Isla Vista virus; TULV, Tula virus; LANV, Laguna Negra virus; ANDV, Andes virus; SNV, Sin Nombre virus; NYV, New York virus.

Blood collected from vole B41 was positive for hantavirus-specific antibodies (indirect fluorescent antibody test that used Puumala antigen) (*8*), suggesting cross-reactivity, as would be expected for Arvicolinae-associated hantaviruses. Hantavirus RNA was detected in the kidneys but not the liver of vole B41 and not in the lungs, liver, or kidneys of the 7 other field voles. Degenerate cytochrome B gene PCR and sequencing (*9*) were used to confirm the morphologic identification of the field voles (B41 CytB GenBank accession no. KC222031). 

The nucleotide and amino acid sequence divergences between B41 and the most related hantaviruses correspond to that typically found between hantavirus species (*5*). The phylogenetic analyses further support B41 as a distinct hantavirus. Thus, we propose to name this novel virus Tatenale virus, reflecting the medieval name of its place of origin.

*M. agrestis* voles, among the most numerous mammals in mainland Britain, have not been shown to be primary carriers of a specific hantavirus, although recent studies suggest that they might be involved in the maintenance of Tula virus in Germany (*10*). Further surveillance is needed to confirm that *M. agrestis* voles are the reservoir hosts of Tatenale virus, provide an estimate of virus prevalence, and determine zoonotic risk. Current knowledge of other *Microtus* vole–borne hantaviruses suggests that although they might infect humans, their pathogenic potential is generally low (*1*). Future work will involve attempts to isolate Tatenale virus and generate its full-genome sequence.

Because hantavirus diseases have such broad clinical features, many cases among humans in the United Kingdom might be misdiagnosed. The confirmation of a novel hantavirus in indigenous wildlife in the United Kingdom might promote inclusion of hantavirus infection in the differential diagnosis for patients with acute renal failure, undiagnosed febrile illness, and exposure to rodents (*4*).

Technical Appendix Location of wild field vole (B41) trapped in August 2011 within United Kingdom, and similarity (% identity) of partial small and large segment sequences in virus from this field vole with other hantaviruses.
