# Supplementary material for: Novel Hantavirus in Wildlife, United Kingdom
Source: Emerg Infect Dis. 2013 Apr;19(4):673–5. doi: 10.3201/eid1904.121057 (PMC3647411; doi:10.3201/eid1904.121057)
Supplement: Technical Appendix — Location of wild field vole (B41) trapped in August 2011 within United Kingdom, and similarity (% identity) of partial small and large segment sequences in virus from this field vole with other hantaviruses. [file 12-1057-Techapp-s1.pdf]

Article DOI: http://dx.doi.org/10.3201/eid1904.121057

Novel Hantavirus in Wildlife, United Kingdom

Technical Appendix

Table. Similarity (% identity) of B41 partial S and L segment sequences with those of other hantaviruses*†‡

	S segment		L segment	
Hantavirus	nt	aa	nt	aa
TOPV	78.8	86.3	n/a	n/a
Fusong	75.0	80.9	77.2	80.0
KHAV	74.7	85.5	n/a	n/a
PUUV	73.7	80.2	76.6	84.8
HOKV	73.5	79.4	76.9	84.8
PHV	68.7	73.3	77.5	88.0
TULV	65.7	66.4	77.5	86.4
SNV	58.1	53.4	71.9	78.4
SWSV	56.3	48.1	68.0	70.4
ANDV	55.8	51.9	72.2	81.6
DOBV	52.8	42.7	63.3	66.4
SEOV	52.8	42.3	63.6	68.8
SAAV	51.8	43.5	63.9	65.6
HTNV	51.5	41.2	66.9	69.6
TPMV	49.2	41.2	63.9	63.2
MGB/1209	n/a	n/a	65.0	62.4

*S, small; L, large; TOPV, Topografov virus (AJ011646); Fusong (EU072481 and FJ170807); KHAV, Khabarovsk (U35255); PUUV, Puumala virus (M32750 and M63194); HOKV, Hokkaido virus (AB675463 and AB675455); PHV, Prospect Hill virus (M34011 and EF646763); TULV, Tula virus (NC005227 and NC005226); SNV, Sin Nombre virus (NC005216 and L37901); SWSV, Seewis virus (GQ293136 and EF636026); ANDV, Andes virus (AF291702 and AF291704); DOBV, Dobrava-Belgrade (AY961615 and GU904039); SEOV, Seoul virus (AY273791 and X56492); SAAV, Saaremaa virus (AJ616854 and AJ410618); HTNV, Hantaan virus (NC005218 and NC005222); TPMV, Thottapalayam virus (AY526097 and NC010707); MGB/1209, Magboi/1209 virus (JN037851); n/a, sequence not available.

†396 nucleotides (nt) of the S segment (positions 620-1015), and 371 nt of the L segment (positions 2962-3332) and the deduced amino acid (aa) sequences (131 aa, position 194-324 of the nucleocapsid protein; 123 aa, position 976-1098 of the viral RNA-dependent RNA polymerase) have been compared using MegAlign (Lasergene DNAStar). Fragment positions were defined according to complete sequences of PUUV strain CG1820.

‡ Partial L sequences obtained following Klempa et al 2006 (5). Partial S sequences obtained using the following primers in the reverse transcription and the first round of PCR: forward (SF490) AARGANAAYAARGGNACN and reverse (SR1157) YTGDATHCCCATNGAYTG. Nested PCR followed with primers: forward (SF604) ATGAARGCNGADGARHTNACN, and reverse (SR1061) CATDATNGTRTTHCTCATRTC.

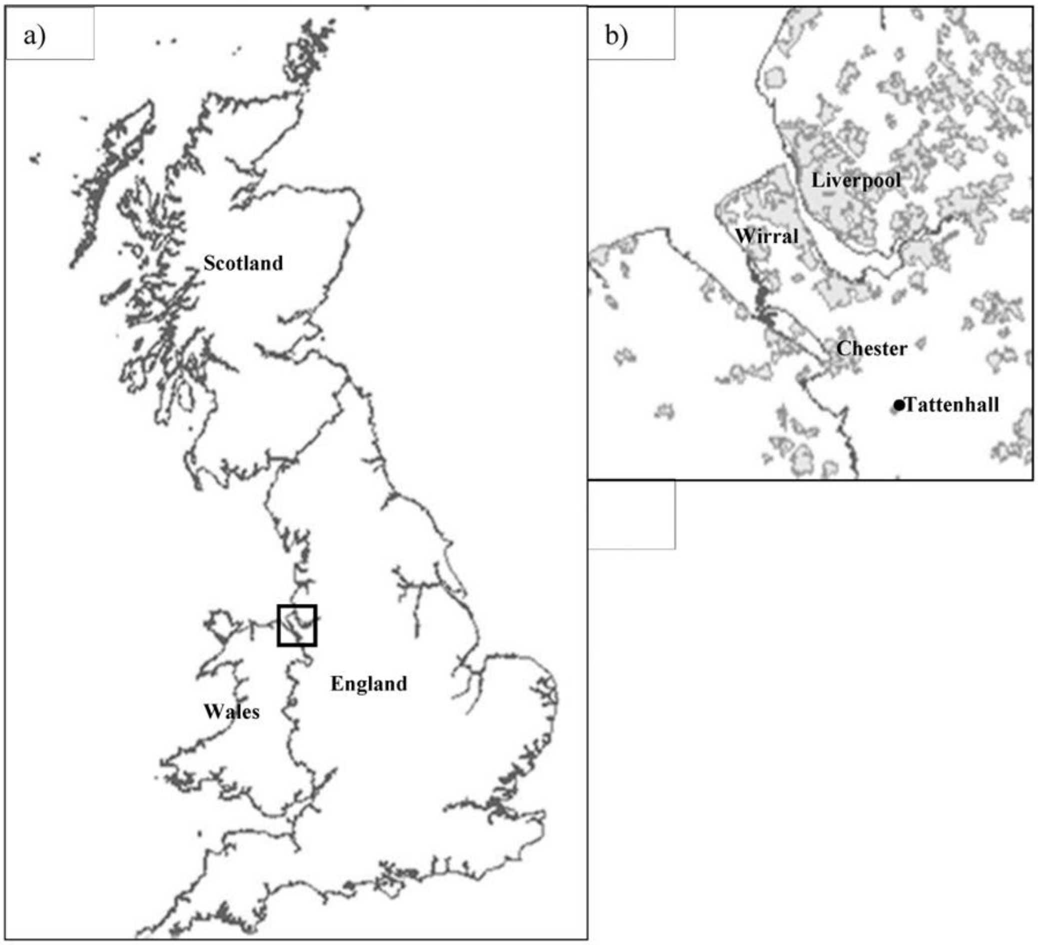

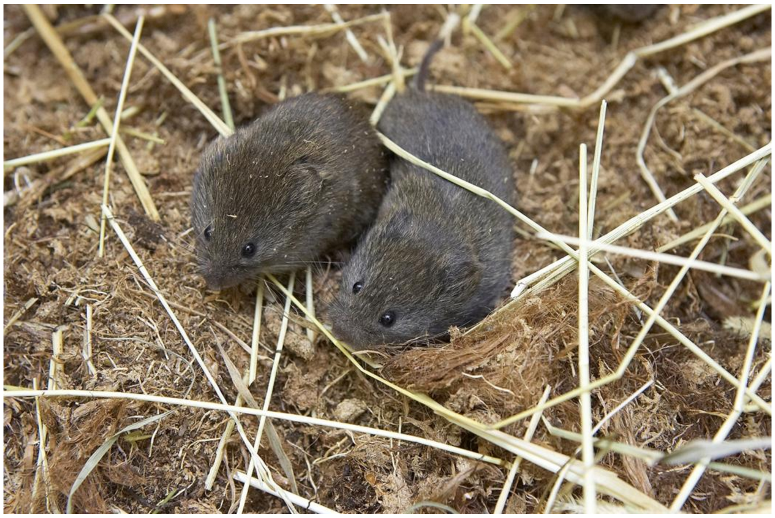

Figure. Location of wild field vole (B41) trapped in August 2011 within United Kingdom (A) and northwestern England (B) (urban areas shown in gray). Image shows field voles (*Microtus agrestis*); image courtesy of E. Oksanen.
